# Factors Associated With Ruminative Thinking in Individuals With Gender Dysphoria

**DOI:** 10.3389/fpsyt.2021.602293

**Published:** 2021-05-25

**Authors:** Dhiordan Cardoso Silva, Leonardo Romeira Salati, Anna Paula Villas-Bôas, Karine Schwarz, Anna Martha Fontanari, Bianca Soll, Angelo Brandelli Costa, Vania Hirakata, Maiko Schneider, Maria Inês Rodrigues Lobato

**Affiliations:** ^1^Transdisciplinary Gender Identity Program, Hospital de Clínicas de Porto Alegre, Porto Alegre, Brazil; ^2^Graduate Program in Psychiatry and Behavioral Sciences, Universidade Federal do Rio Grande do Sul, Porto Alegre, Brazil; ^3^Graduate Program in Psychology, Pontifical Catholic University of Rio Grande do Sul, Porto Alegre, Brazil; ^4^Biostatistic Unit, Hospital de Clínicas de Porto Alegre, Porto Alegre, Brazil; ^5^Departments of Psychiatry and Behavioral Neurosciences, McMaster University, Hamilton, ON, Canada; ^6^Youth Wellness Center, St Joseph Healthcare Hamilton, Hamilton, ON, Canada

**Keywords:** ruminative thinking, transgender persons, gender dysphoria, depression, anxiety, stress

## Abstract

This study aimed to examine psychosocial factors and medical history as well as symptoms of depression, anxiety, and stress associated with ruminative thinking in transgender people with gender dysphoria (GD) before undergoing gender affirmation surgery (GAS). This study evaluated 189 participants with GD (111 trans women and 78 trans men) from a specialized service for GAS in southern Brazil. Semi-structured interviews were conducted, and participants were asked to complete self-report questionnaires. We recovered participants' sociodemographic and psychosocial data (e.g., history of sexual abuse, expulsion from home, and history of drug use) and data regarding their clinical history (e.g., medication, history of suicidal ideation and attempted suicide, and HIV status). Further, we implemented the Depression, Anxiety and Stress Scale (DASS-21) to examine participants' psychological state, as well as the Ruminative Response Scale (RRS) to assess ruminative thinking, which includes brooding and reflection. The predictor variables were those that exhibited a minimum level of significance of *p* ≤ 0.05 in multivariate linear regression. The ruminative thinking scores for trans women were higher than those of trans men (Brooding *p* = 0.014; Reflection *p* = 0.052).In the multivariate model, suicidal ideation, moderate depression, and severe/very severe anxiety were associated with both brooding and reflection. Feminine gender identity and stress symptoms moderated only brooding, while anxiety symptoms moderated only reflection. Our findings show that trans women had the highest ruminative thinking scores, and that depression, anxiety, stress, and suicidal ideation were associated with ruminative thinking in total sample. Psychological symptoms should be examined in the context of gender affirmation surgery to minimize the possibility of adverse mental health outcomes. Follow-up studies are required to measure ruminative thinking levels more accurately and to identify its predictors.

## Introduction

Ruminative thinking (RT) is defined as a repetitive thinking pattern causing adverse emotional responses that impair the individual's use of psychological coping strategies (1). It can also be described as the persistent tendency to harbor self-centered thoughts, intrinsic in people in general, but which can become dangerous to psychological health and can accompany different clinical conditions (2, 3). The presence of RT can have consequences for cognitive function (decision-making and memory) (4) and can cause the production and/or exacerbation of psychopathological symptoms and clinical conditions such as anxiety and depression (5–8).

In preexisting literature, RT has been highlighted as a marker of poor prognosis in different clinical conditions, especially when the tendency to ruminate keep a certain stability and a considerable degree of intensity (5), as seen in cases of depression, personality disorders, anxiety disorders, suicidal ideation, and situations of sexual violence, among others (3, 9–14). Silveira et al. (15), analyzed RT patterns in individuals diagnosed with schizophrenia, schizoaffective disorder, bipolar disorder, depressive disorder and anxiety disorder and showed rumination as a poorly adaptable coping style associated with worry, distress, disease severity and status socioeconomic.

Gender dysphoria (GD) (16) is defined as an individual's feeling of discomfort between their biological sex and the social gender attributed to them. This feeling is accompanied by a strong desire to change their body and/or attire to better reflect their gender identity. In a previous study published by our group, Mueller et al. (17) carried out a cross-sectional analysis of trans women diagnosed with GD that sought to compare the levels of RT in three groups of women. The first group included trans women at the beginning of treatment, the second group included trans women who had undergone 1 year of clinical care and mental health, while the third group included women who had already undergone gender affirmation surgery (GAS). We found that RT levels were lower in the third group, highlighting the positive impact of the surgical approach on that group's GD. For this study, we tried to expand the sample of participants and test another hypothesis of association.

We try to understand RT from an alternative multidimensional perspective (implementing the Domain of Negative Valuation Systems of the Research Domain Criteria) (18) to examine different diagnoses and outcomes regarding mental health. Independently of gender identity, all people have levels of RT, being cisgender or transgender. In order to broaden the understanding of RT in individuals with GD, our study expanded the sample and sought to test the associations of RT with psychosocial factors, acute symptoms of anxiety, depression and stress from a sample with GD from a hospital service in Southern Brazil.

## Materials and Methods

This was a cross-sectional study, with non-probabilistic sampling, carried out with the Transdisciplinar Gender Identity Program (PROTIG). PROTIG is a reference service for performing GD related surgical procedures in Brazil, conforming to the country's public health laws and regulations (19). In the beginning of the program, individuals enter a GD evaluation period of up to 3 months, where they receive monthly consultations. Once the diagnosis is confirmed, the patient is referred to psychological support groups until they undergo GAS.

The study was registered and approved by the Research Ethics Committee of the Hospital de Clínicas de Porto Alegre (CAAE: 73303717.2.0000.5327). After receiving an invitation to participate in this study, the participants signed an informed consent form. For this study, we followed the Strengthening the Reporting of Observational Studies in Epidemiology (STROBE) guidelines (20).

### Participants

We evaluated 189 transgender individuals, including 111 trans women (assigned male at birth) and 78 trans men (assigned female at birth) who met the following eligibility criteria: having undergone clinical evaluation, having received a GD diagnosis (16), having no history of GAS, having no history of drug use in the past 2 weeks, and having the necessary comprehension skills to respond to the interview.

### Materials

We implemented a semi-structured interview protocol to obtain participants' psychosocial, sociodemographic, and clinical data. *Cross-dressing age* was defined as the age at which the individual began to habitually dress in clothes assigned to the opposite sex as part of a performance. *Social transition age* was defined as the age at which the individual socially revealed their gender identity. The medical record was consulted to collect additional information on participants' childhood and vulnerabilities. The Ruminative Response Scale (RRS) 10-item revised version (9, 21, 22) is a scale comprising 10 items scored on a four-point Likert scale ranging from 1 (*almost never*) to 4 (*almost always*), and was used to assess RT, with higher scores indicating a higher degree of ruminative symptoms. The RRS identified two subtypes of rumination: brooding (B) and reflection (R). Brooding refers to experiencing negative mood symptoms and is strongly associated with short- and long-term depression (example items include “What am I doing to deserve this,” “why do I always react this way?”). Reflection refers to individuals' general self-reflective tendency to focus on trying to solve problems; it is related to short-term depression (example items: “Analyse recent events to try to understand why you are depressed” and “Go someplace alone to think about your feelings”) (7, 21). Each subtype consisted of five items scored on a Likert scale ranging from 1 (*almost never*) to 4 (*almost always*). For evaluating ruminative style, B and R were used as continuous variables (raw scores). The Depression, Anxiety and Stress Scale (DASS-21) (23, 24) is a four-point Likert scale, with 21 items that aimed to assess the individual's depression, anxiety, and stress symptoms in the past week (acute symptoms) through three subscales. The scale assessed responders' interpretation of events as well as their physiological and psychological responses (25). Total scores were obtained by adding the scores of the seven items for each of the three subscales. The scale provided three notes, one per subscale, with a minimum of 0 and a maximum of 21. For this study, we grouped the scores as follows: depression (normal/mild = 0–13; moderate = 14–20; severe/extremely severe = 21+), anxiety (normal/mild = 0–9; moderate = 10–14; severe/extremely severe = 15+) and stress (normal/mild = 0–18; moderate = 19–25; severe/extremely severe = 26+).

### Statistical Analysis

Results are presented as descriptive statistics through absolute and relative frequencies for categorical variables, means, and standard deviations for quantitative variables. The normality of continuous variables was tested using the Shapiro Walk test. Statistical analyses were performed using SPSS software (26).

Bivariate analyses were performed using simple linear regression. The independent variables that presented results of *p* < 0.20 were included in the multiple linear regression. The following variables were considered: gender identity:trans women (ref category: trans men), age, sexual abuse (ref category: yes), expelled from home (ref category: yes), suicidal ideation (ref category: yes), suicide attempt (ref category: yes), moderate depression (ref category: normal/mild and severe/extremely severe), moderate and severe/very severe anxiety (ref category: normal/mild), and moderate stress (ref category: moderate and severe/extremely severe). The age of expulsion from home was not included in the multivariate model, as some participants provided limited information in this regard.

Predictive variables were considered to be those with a minimum level of significance of *p* ≤ 0.05(B = gender identity, suicidal ideation, moderate depression, severe/very severe anxiety, moderate stress; R = suicidal ideation, moderate depression, moderate anxiety, and severe/very severe anxiety). Multicollinearity was tested using variance inflation factor (VIF) statistics. Since the variables depression, anxiety, and stress (DASS-21) presented multicollinearity, we decided to conduct the analyses in separate models to observe how they behaved independently in the multivariate model with RRS. The results were presented as non-standardized coefficients and a 95% confidence interval (95% CI).

## Results

Descriptive statistics of demographic and clinical data are shown in Table 1. Most of our sample was composed of trans women (58.7%; *n* = 111), with the average age being 29.03 (SD = 8.90) years. Considering schooling, about 66.7% (*n* = 126) of participants has school graduate or +. Most participants (85.2%) reported not being in a stable loving relationship. Regarding issues of transition and affirmation of gender identity, the average of cross-dressing age was 6.62 (SD = 3.17) years and 19.8 (SD = 6.21) years waste average social transition age. Regarding clinical issues, 24 trans women and 1 trans man were HIV positive, while 35.5% (*n* = 65) reported a history of drug use. It is noteworthy that 22.9% (*n* = 41) participants reported having experienced sexual abuse during childhood, while 13.4% (*n* = 24) reported having engaged in sex work. In all, 12 participants (6.5%; nine women and three men) reported having been expelled from home with an average age of 16.55 (SD = 4.05) years. Regarding psychosocial clinical issues, almost half the participants, about 46.4% (*n* = 51) trans women and 47.4% (*n* = 37) trans men, reported having experienced suicidal ideation, while 31.6% (*n* = 59) of the total sample had attempted suicide at some point in their life. For the variable “time in service” before to GAS, we classified the sample into three groups: without clinical care in therapeutic groups (not started group), ≤ 1 year of clinical care in the service, >1 year of clinical care.

**Table 1:** Sociodemographic, psychosocial, and clinical characterization of the sample.

	***N* valid**	**Total sample**	**Trans women**	**Trans men**
Gender identity, *N* (%)	189	189	111 (58.7)	78 (41.3)
Age, M (SD)	189	29.0 (9.0)	29.5 (8.9)	28.4 (8.8)
Schooling, *N* (%)	189			
Incomplete fund		16 (8.5)	11 (9.9)	5 (6.4)
Complete fund		47 (24.9)	32 (28.8)	15 (19.2)
School graduate or +		126 (66.7)	68 (61.3)	58 (74.4)
Relationship Status, *N* (%)	189			
Dating a person/with exclusive romantic relationship		28 (14.8)	12 (10.8)	16 (20.5)
Not dating/no exclusive romantic relationship		161 (85.2)	99 (89.2)	62 (79.5)
Age cross-dressing, M (SD)	43	6.6 (3.2)	6.8 (4.0)	6.4 (2.1)
Age social transition, M (SD)	183	19.8 (6.2)	19.7 (6.6)	20.0 (5.6)
HIV, *N* (%)	189	25 (13.2)	24 (21.6)	1 (1.3)
Sexual abuse, *N* (%)	179	41 (22.9)	23 (22.8)	18 (23.1)
Sex work, *N* (%)	179	24 (13.4)	23 (22.8)	1 (1.3)
Suicidal ideation, *N* (%)	188	88 (46.8)	51 (46.4)	37 (47.4)
Suicide attempt, *N* (%)	187	59 (31.6)	34 (31.2)	25 (32.1)
Expelled from home, *N* (%)	179	12 (6.5)	9 (8.3)	3 (3.8)
Age Expelled, M (SD)	11	16.6 (4.1)	16.9 (4.8)	15.7 (1.2)
Illicit drug use past, *N* (%)	183	65 (35.5)	34 (32.1)	31 (40.3)
Time in service	189			
≤ 1 year		22 (11.6)	14 (12.6)	8 (10.3)
>1 year		62 (32.8)	34 (30.6)	28 (35.9)
Not started group		105 (55.6)	63 (56.8)	42 (53.8)
Depression DASS-21, *N* (%)	177			
Normal		153 (86.4)	91 (83.5)	62 (91.2)
Moderate		21 (11.9)	15 (13.8)	6 (8.8)
Severe/very severe		3 (1.7)	3 (2.8)	–
Anxiety DASS-21, *N* (%)	177			
Normal		141 (79.7)	82 (75.2)	59 (86.8)
Moderate		22 (12.4)	16 (14.7)	6 (8.8)
Severe/very severe		14 (7.9)	11 (10.1)	3 (4.4)
Stress DASS-21, *N* (%)	177			
Normal		170 (96)	104 (95.4)	66 (97.1)
Moderate		7 (4)	5 (4.3)	2 (2.9)
Severe/very severe		–	–	–
RRS, M (SD)				
Brooding	189	11.7 (3.9)	12.2 (3.9)	10.8 (3.7)
Reflexion	189	11.8 (3.9)	12.3 (4.0)	11.1 (3.9)
Total	189	23.4 (7.1)	24.5 (7.2)	21.9 (6.8)

*N, sample; %, percentage; M, mean; SD, standard deviation; DASS-21, depression, anxiety and stress scale; RRS, ruminative response scale*.

The statistical results of the bivariate model are specified in Table 2. In the bivariate model, trans women had higher RT scores (B: *p* = 0.014; R: *p* = 0.052). Brooding was significantly associated with age and being expelled from home (*p* = 0.087 and *p* = 0.122, respectively). History of sexual abuse was associated with B (*p* = 0.031) and R (*p* = 0.075), suicidal ideation (B and R *p* < 0.001), suicide attempt (B *p* = 0.001; R: *p* = 0.002), symptoms of moderate depression (B and R *p* < 0.001), moderate anxiety (B *p* = 0.007; R: *p* = 0.002), moderate stress (B *p* = 0.01; R *p* = 0.01) and severe/very severe stress (B *p* < 0.001; R *p* = 0.04). Regarding participants' psychosocial characteristics, despite their apparent clinical relevance, these factors lost statistical power as predictors of RT in the multivariate model.

**Table 2:** Bivariate model of brooding and reflexion associated with psychosocial, clinical and symptomatological variables (*n* = 189).

**Variable**	**Brooding (*****n*** **=** **189)**		**Reflexion (*****n*** **=** **189)**		**RSS total (*****n*** **=** **189)**	
	**b (95% CI)**	**Sig**	**b (95% CI)**	**Sig**	**b (95% CI)**	**Sig**
Trans women	1.38 (0.28; 2.48)	0.014	1.12 (−0.01; 2.25)	0.052	1.12 (−0.01; 2.25)	0.052
Age	−0.05 (−0.12; 0.01)	0.087	−0.04 (−0.10; 0.02)	0.229	−0.09 (−0.20; 0.02)	0.109
Age cross-dressing	0.19 (−0.13; 0.52)	0.240	−0.13 (−0.48; 0.22)	0.474	0.06 (−0.53; 0.65)	0.831
Age social transition	0.02 (−0.07; 0.11)	0.714	0.03 (−0.06; 0.12)	0.535	0.05 (−0.12; 0.21)	0.587
Schooling - fund incomplete	0.47 (−1.54; 2.48)	0.648	0.75 (−1.30; 2.80)	0.475	0.75 (−1.30; 2.79)	0.475
Schooling - fund complete	0.34 (−0.96; 1.64)	0.606	0.18 (−1.14; 1.50)	0.787	0.18 (−1.14; 1.50)	0.787
With affective relationship	0.31 (−1.25; 1.88)	0.696	0.73 (−0.84; 2.30)	0.362	0.731 (−0.84; 2.30)	0.362
Sex work	0.22 (−1.43; 1.88)	0.791	−0.42 (−2.10; 1.27)	0.629	−0.41 (−2.10; 1.27)	0.629
Sexual abuse	1.46 (0.14; 2.79)	0.031	1.23 (−0.12; 2.59)	0.075	1.23 (−0.12; 2.58)	0.075
Expelled from home	1.78 (−0.47; 4.04)	0.122	−0.25 (−2.56; 2.06)	0.832	−0.25 (−2.56; 2.06)	0.832
Illicit drug use past	−0.01 (−1.18; 1.17)	0.992	0.18 (−1.01; 1.38)	0.763	0.18 (−1.01; 1.38)	0.763
HIV diagnosis	−0.43 (−2.06; 1.20)	0.602	−0.05 (−1.71; 1.62)	0.958	−0.04 (−1.70; 1.62)	0.958
Suicidal ideation	2.33 (1.27; 3.39)	0.001	2.40 (1.32; 3.48)	0.001	2.39 (1.32; 3.48)	0.000
Suicide attempt	1.94 (0.80; 3.09)	0.001	1.91 (0.71; 3.10)	0.002	1.90 (0.71; 3.09)	0.002
≤ 1 year in Protig	0.85 (−0.92; 2.62)	0.347	0.66 (−1.15; 2.47)	0.473	0.66 (−1.14; 2.47)	0.473
>1 year in Porting	−0.57 (−1.77; 0.64)	0.359	−0.35 (−1.59; 0.88)	0.574	−0.35 (−1.59; 0.88)	0.574
Depression moderate	3.92 (2.30; 5.54)	0.001	3.03 (1.33; 4.73)	0.001	3.02 (1.32; 4.72)	0.000
Depression severe/very severe	−3.13 (−7.19; 0.93)	0.131	−3.35 (−7.61; 0.91)	0.123	−3.35 (−7.61; 0.91)	0.123
Anxiety moderate	2.21 (0.62; 3.81)	0.007	2.70 (1.01; 4.39)	0.002	2.70 (1.01; 4.38)	0.002
Anxiety severe/very severe	4.55 (2.59; 6.50)	0.001	2.16 (0.10; 4.22)	0.040	2.16 (0.10; 4.22)	0.040
Stress moderate	3.70 (0.88; 6.52)	0.010	3.73 (0.85; 6.62)	0.011	3.73 (0.84; 6.61)	0.011
Stress severe/very severe	–	–	–	–	–	–

Finally, in the final multivariate model, trans women's brooding was associated with depression and stress (*p* = 0.028; *p* = 0.029, respectively). Suicidal ideation was associated with depression (B *p* = 0.033; R *p* = 0.014), anxiety (B and R *p* = 0.008), and stress (B *p* = 0.034; R *p* = 0.015). Moderate depression (B *p* < 0.001; R *p* = 0.004), moderate anxiety (R *p* = 0.012), severe/very severe anxiety (B *p* < 0.001; R *p* = 0.038), and moderate stress (B *p* = 0.032) were predictive factors for RT (Table 3).

**Table 3:** Multivariate model associated with brooding and reflexion (*n* = 167).

	**Brooding (*****n*** **=** **167)**		**Reflexion (*****n*** **=** **167)**		**RSS total (*****n*** **=** **167)**	
	**b**	**Sig**.	**b**	**Sig**.	**b**	**Sig**.
**Depression DASS-21**
Trans women	1.19 (0.13; 2.25)	0.028	1 (−0.11; 2.11)	0.077	2.16 (0.25; 4.07)	0.027
Age	−0.05 (−0.11; 0.01)	0.114	–	–	–	–
Suicidal ideation	1.43 (0.12; 2.74)	0.033	1.71 (0.35; 3.07)	0.014	3.31 (0.97; 5.66)	0.006
Suicide attempt	0.18 (−1.19; 1.56)	0.793	0.28 (−1.17; 1.72)	0.706	0.53 (−1.96; 3.02)	0.675
Sexual abuse	0.63 (−0.66; 1.92)	0.339	0.27 (−1.08; 1.62)	0.697	0.77 (−1.56; 3.10)	0.518
Expelled from home	0.85 (−1.16; 2.86)	0.408	–	–	–	–
Depression moderate	3.19 (1.48; 4.9)	0.001	2.59 (0.81; 4.38)	0.004	5.81 (2.73; 8.89)	0.000
Depression severe/very severe	−2.1 (−6.87; 2.67)	0.388	−1.22 (−6.23; 3.8)	0.635	−3.29 (−11.95; 5.37)	0.456
**Anxiety DASS-21**
Trans women	0.9 (−0.14; 1.94)	0.088	0.8 (−0.31; 1.91)	0.157	1.68 (−0.22; 3.57)	0.083
Age	−0.04 (−0.1; 0.02)	0.191	–	–	–	–
Suicidal ideation	1.72 (0.46; 2.99)	0.008	1.83 (0.48; 3.19)	0.008	3.70 (1.39; 6.01)	0.002
Suicide attempt	0.12 (−1.22; 1.45)	0.861	0.26 (−1.18; 1.7)	0.725	0.43 (−2.02; 2.88)	0.729
Sexual abuse	0.26 (−1.02; 1.53)	0.695	−0.02 (−1.4; 1.36)	0.977	0.10 (−2.24; 2.44)	0.933
Expelled from home	0.7 (−1.26; 2.65)	0.485	–	–	–	–
Anxiety moderate	1.54 (−0.02; 3.1)	0.052	2.13 (0.46; 3.8)	0.012	3.80 (0.96; 6.64)	0.009
Anxiety severe/very severe	5.05 (3.06; 7.05)	0.001	2.28 (0.13; 4.42)	0.038	7.41 (3.75; 11.06)	0.000
**Stress DASS-21**
Trans women	1.21 (0.12; 2.3)	0.029	1.04 (−0.08; 2.15)	0.069	2.23 (0.28; 4.18)	0.025
Age	−0.04 (−0.11; 0.02)	0.148	–	–	–	
Suicidal ideation	1.45 (0.11; 2.8)	0.034	1.71 (0.33; 3.08)	0.015	3.32 (0.92; 5.72)	0.007
Suicide attempt	0.54 (−0.86; 1.94)	0.453	0.56 (−0.89; 2.01)	0.447	1.17 (−1.35; 3.70)	0.363
Sexual abuse	0.66 (−0.67; 2)	0.329	0.29 (−1.09; 1.66)	0.68	0.83 (−1.57; 3.23)	0.497
Expelled from home	1.17 (−0.89; 3.22)	0.267	–	–	–	–
Stress moderate	3.14 (0.28; 6)	0.032	2.82 (−0.15; 5.79)	0.062	6.07 (0.89; 11.25)	0.022
Stress severe/very severe	–	–	–	–	–	–

## Discussion

Our study identified RT in transgender participants with GD. By conducting interviews and utilizing self-reported instruments, we found that in GD, RT can expose the individual to mental health symptoms. Although the sample included individuals with or without a previous diagnosis of anxiety disorder and/or depressive disorder, the emphasis of the study was to examine the association of RT with acute psychological symptoms and psychosocial factors.

Our findings revealed a greater tendency for trans women to ruminate, similar to cisgender women from other studies that evaluated the association between RT and gender where have revealed that adult women have a predisposition for RT (27–32) and that this pattern could be associated with a greater number of depressive episodes and chronic depression (7, 28, 33). In 1987, Nolen-Hoeksema (27) proposed that the prognostic difference between depressed women and men could be explained by biological sexual differences, differences in social roles, and “learned female helplessness,” perpetuating existing gender stereotypes according to which activity and emotional repression are characterized as male traits while emotionality and passivity are seen as female traits.

Conversely, Broderick and Kortelan studied the link between rumination, depression, and gender roles in children 9 to 12 years old (29). They observed that regardless of the gender individuals were assigned at birth, those who identified with female characteristics showed more RT traits, suggesting that the socialization processes of girls and women (rather than biological sex) are a risk factor for the development of RT (34).

Watkins and Nolen-Hoeksema suggested that the social construction of the “subordinate role of women” (learned passivity) (reflection of a society with patriarchal beliefs with social arrangements of privileges and power given to men) and the development of self-centered functioning (with a focus on observing one's emotions) generate an increase in “tension and attempted emotional control in the face of stressful situations” that puts individuals at a greater risk of developing RT (6).

In addition to RT being harmful to people's mental health in general, the role of stress in the production of depressive symptoms has been widely acknowledged by researchers. Mezo and Baker describe rumination as an important moderator in the relationship between stress and depression, and highlight the importance of assessing the type of stress (25). They suggest the integration of a rumination style in response to stressful events (pathophysiological model) with the negative cognitive processing, as a risk trigger for depression (25, 35).

Our study found, as a predictive factor of RT, three categories of acute symptoms: anxiety, depression, and stress (measured using the DASS-21). The use of the DASS-21 aimed to understand individuals selective actions in the face of symptoms of irritability, generalization of tension, concerns, and sadness, without necessarily establishing a descriptive characterization of the triggering events themselves. It is interesting to note that although most of the participants had normal DASS-21 scores and were in active care in a specialized hospital service, we identified individuals with moderate and severe scores on the DASS-21, which suggests a failure in the continuous care offered, perhaps because it was excessively focused on preparing them for GAS.

The extremely stressful psychosocial circumstances experienced by individuals with GD (including physical/verbal violence and social exclusion), in addition to possible temperamental predispositions, make them vulnerable to developing maladaptive emotional abilities, such as RT, anxiety, and depression (36–39).

Our results indicate that 47% of participants had experienced suicidal ideation and 31% had attempted suicide at some point in the past. Suicidal ideation was considerably associated with RT, similar to other studies (12, 40–42). Our findings suggest that it is essential to examine RT's association with suicidal ideation among individuals with GD, as it is conducive to poor mental health outcomes. Wang et al. reported similar results when analyzing RT as a mediating component of negative life events and suicidal ideation, in a sample of cisgender university students (43).

We believe that this study's cross-sectional design did not provide the opportunity to reveal a subjective representative finding of clinical improvement related to the time of treatment. In a previous study, Mueller et al. (17) observed that RT levels were significantly lower the longer the treatment lasted when comparing three groups of trans women in different stages of specialized care (from initial evaluation until after GAS).

RT was not associated with positive HIV status, history of drug use, cross-dressing age, or social transition age. Therefore, individuals' age at the time of the affirmation of their gender identity did not seem to influence RT. The process of construction and social transition of gender identity is complex, generating anxiety and social repercussions that vary depending on the cultural setting. For Brazilian transgender individuals, this is a long process involving self-reflection regarding the formation of their identity in an adverse *cisnormative* social context where insufficient access to health services, insufficient professionalization, social marginalization, as well as physical and psychological illness are commonplace.

## Conclusion

Our study is the first to analyze the psychosocial, sociodemographic, and clinical factors associated with RT in a transgender population with GD participating in a specialized hospital program for GAS. The mechanisms underlying RT are important indicators to be taken into consideration during clinical evaluation and in the development of preventive strategies to improve trans individuals' mental health regarding GD.

Moreover, our study contributes to the literature on RT and its associated factors among individuals with GD within the context of GAS. It provides helpful insights for teams of mental health professionals regarding the planning of therapeutic actions to prevent RT (especially in individuals with a history of suicidal ideation, attempted suicide, depression, anxiety, and stress). We emphasize the importance of expanding specialized mental health services in the health of trans populations, especially in the prevention and treatment of psychiatric disorders, in addition to preventing suicide and making them less vulnerable to aversive social situations. Having said that, this study has some limitations in terms of multiple analysis, even though it was not the methodological interest of this study, it was important to insert a control group (i.e., depression, anxiety and healthy people) and expand the, sample size. Thus, future studies should consider the possibility of expanding their samples to cover specialized GAS services, inserting a control group and verifying the comparison between groups with less and more rumination in individuals with GD. Therefore, our findings highlight the importance of carrying out studies with a longitudinal design that include symptomatic assessment of RT and larger samples of individuals in specialized services aimed at performing GAS.

## Data Availability Statement

The original contributions presented in the study are included in the supplementary article/material, further inquiries may be directed to the corresponding author(s).

## Ethics Statement

The studies involving human participants were reviewed and approved by Research Ethics Committee of the Hospital de Clínicas de Porto Alegre (HCPA) (protocol no. 170639). The patients/participants provided their written informed consent to participate in this study.

## Author Contributions

DS designed the study, wrote the protocol, was responsible for the analysis and interpretation of data, drafting the article, and approving this version of the manuscript. LS, AV-B, KS, AF, BS, and AC participated in data collection and approving this version of the manuscript. VH, MS, and ML were responsible for study design and interpretation of data, drafting the article, and approving this version of the manuscript. All authors contributed to the article and approved the submitted version.

## Conflict of Interest

The authors declare that the research was conducted in the absence of any commercial or financial relationships that could be construed as a potential conflict of interest. The reviewer AA declared a shared affiliation, though no other collaboration, with one of the authors AC, to the handling Editor. The reviewer ES declared a shared affiliation, though no other collaboration, with several of the authors DS and ML, to the handling Editor.
